# Burkitt's lymphoma mimicking EBV disease as first sign of vertical HIV infection in an adolescent

**DOI:** 10.1186/1824-7288-36-34

**Published:** 2010-04-23

**Authors:** Veronica Santilli, Nadia Mora, Angela Aquilani, Hyppolite K Tchidjou, Giuseppe Pontrelli, Rita De Vito, Alessandra Lombardi, Stefania Bernardi, Paolo Palma

**Affiliations:** 1 DPUO, University Department of Pediatrics- Children's Hospital Bambino Gesù, Rome, Italy; 2 Unit of Anatomical Pathology- Children's Hospital Bambino Gesù, Rome, Italy; 3 Unit of Haematology- Children's Hospital Bambino Gesù, Rome, Italy

## Abstract

Burkitt's Lymphoma (BL) rarely represents the first clinical manifestation of vertical HIV infection in adolescent in Western Europe. We report the case of a 17 year-old boy with two week history of fever and enlarged cervical lymph nodes firstly misdiagnosed as EBV infection, subsequently diagnosed as Burkitt's Lymphoma and vertical HIV infection.

## Background

Human immunodeficiency virus (HIV)-infected children are at increased risk of developing cancer, particularly in the later stages of acquired immune deficiency syndrome (AIDS) [1]. The introduction of efficacious regimens of antiretroviral treatment has resulted in a substantial and dramatic decrease in AIDS-related opportunistic infections and cancers [1]. However, despite this progress, recent data show as HIV infection itself represents a major risk factor for developing cancers and non-Hodgkin's Lymphoma (NHL) which remains the most frequent malignancy in subjects with AIDS [2].

## Case Report

A 17 years-old boy was admitted with a short history of fever, tonsillitis and monolateral enlarged cervical lymph nodes non responsive to antibiotic therapy with Clarithromycin. Familiar history showed mother's death for lymphoma and remote history was silent with normal growth and neurocognitive development and a single episode of bronchopneumonia at the age of 4 years. Initial clinical examination revealed good general condition except for the enlargement of right side cervical lymph node 3 cm in diameter hard and painful, hepatosplenomegaly, pharyngeal hyperemia. No other alterations were found, even through neurological examination. Biological tests disclosed the following values: haemoglobin 11.9 g/dl; leucocytes 5.78 × 10^3/uL with 39,5% lymphocytes and 50,7% neutrophils; platelets 91 × 10^3/uL; sedimentation rate, 77 mm/h; CRP 1.36 mg/dl; LDH 523 UI/L; GOT 57 UI/L; GPT 41 UI/L [Table 1].

**Table 1:** Patient's haematological data on admission and during follow-up.

	On admission	After 5 months of Chemotherapy and HAART	After 10 months of follow up	After 2 years of follow up	After 3 years of follow up
**Hb (g/dl)**	11.9	11.6	14.6	15.5	16.1

**RBC (×10^6/uI)**	4.38	3.87	4.57	4.91	5.07

**WBC (×10^3/uI)**	5.78	5.21	6.82	8.36	8.25

**Neu (×10^3/uI)**	2.93	2.63	3.41	5.01	5.10

**Lym (×10^3/uI)**	2.20	1.94	2.82	2.51	2.46

**PLT (×10^3/uI)**	91	177	230	221	218

**CRP mg/dl**	1.36	0.25	0.20	< 0.05	0.47

**LDH (UI/L)**	523	441	354	248	286

**GOT (UI/L)**	57	16	22	18	18

**GPT (UI/L)**	41	13	12	12	12

**IgA (mg/dl)**	608	288	284	320	339

**IgG (mg/dl)**	2306	707	1256	1025	933

**IgM (mg/dl)**	440	122	250	145	134

**HIV Viral Load** **(copies/ml)**	118.000	320	<50	<50	<50

**CD4+%/abs** **(cell/uI)**	5%/114	9%/174	11%/298	21%/527	25%/603

**EBV RNA blood/plasma** **(copies/ml)**	0/20.000	0/43.000	113.000/5.400	0/0	0/0

On the basis of clinical and laboratory signs we performed microbiological tests to rule out Epstein-Barr virus (EBV) infection, our first diagnostic suspect. Serology test for EBV showed the presence of anti-EBNA and VCA IgG whereas IgM anti-VCA resulted negative. In parallel, polymerase chain reaction (PCR) detected the presence of 20.000 EBV blood copies/ml. ELISA test for hepatitis A (HAV), hepatitis C (HCV), hepatitis B (HBV), cytomegalovirus (CMV) resulted IgM and IgG negative. Cervical ultrasound showed enlarged right side cervical lymph nodes 4,5 cm in diameter with a loss of hilar echogenic central and hilar architecture, presence of intranodal necrosis, calcification and high peripheral and central perfusion. There were also multiple nodes in the left side of about 1 cm in diameter. Abdominal ultrasound showed hepatosplenomegaly and modest increase of hepatic hilus and retroperitoneal lymph nodes. Mycobacterial infection was excluded by Interferon gamma release assay.

**Figure 1:**
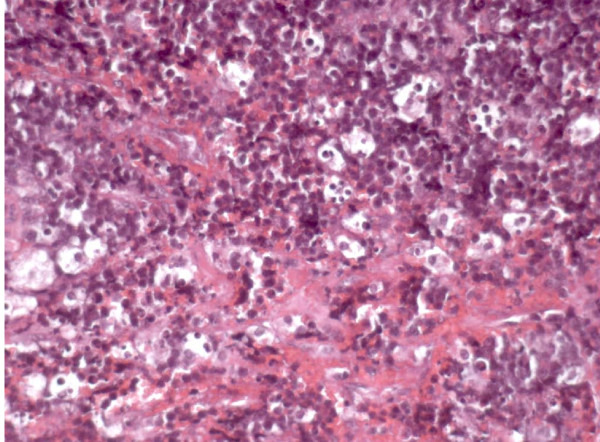
**Hematoxylin and eosin stainig: Hematoxylin and eosin section showing monomorphic population of neoplastic lymphoid cells and large pale macrophages forming the "stars" in the "starry sky" typical of Burkitt Lymphoma (HE 20×)**.

**Figure 2:**
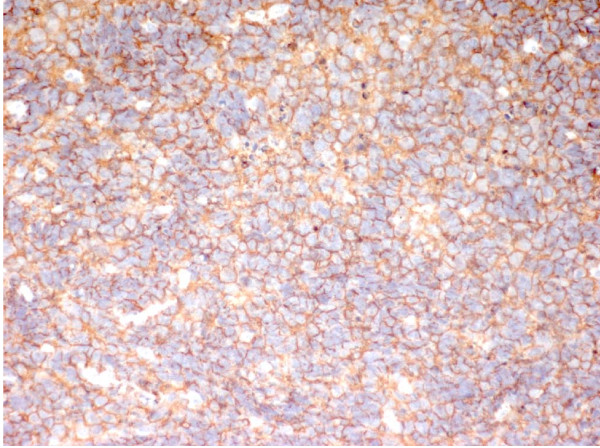
**Immunohistochemical staining: Immunohistochemistry showing tumor cells are positive for CD20 (CD20 20×)**.

**Figure 3:**
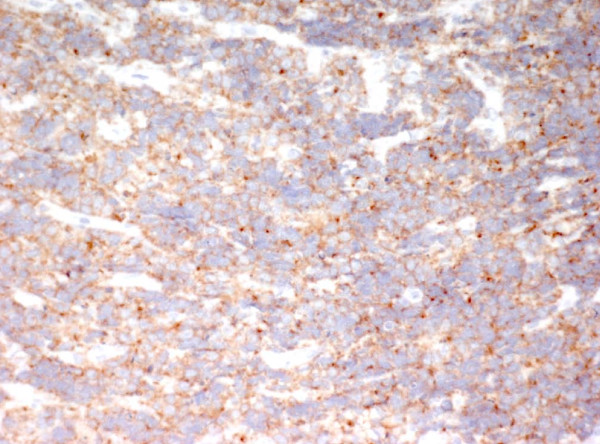
**Immunohistochemical staining: Immunohistochemistry showing tumor cells are positive for CD10 (CD10 20×)**.

Due to the persistence of tonsillopharyngitis and lymphoadenopathy over two weeks of follow-up, the patient underwent open biopsy showing a malignant population of round monomorphic B cells, interspersed with macrophages forming the "stars" in the "starry sky" histology pattern typical of the Burkitt's Lymphoma (BL) [Fig 1]. The malignant cells were strongly positive for CD20, CD79a and CD10 [Fig 2, 3] and T-lymphocyte markers (CD3 and TdT) were negative. Detection of EBV RNA in corresponding tumor tissues was carried out using *in situ *hybridization. In view of the hypergammaglobulinemia (IgA 608 mg/dl, IgG 2306 mg/dl and IgM 440 mg/dl) associated with severe immunodeficiency (CD4 count 114/mm^3^-5%) HIV infection was suspected and confirmed by standard ELISA serology test and quantitative PCR (HIV viral load 118.000 copies/ml). Thus, diagnosis of HIV-related BL was established and subsequently confirmed in his mother by the acquisition of her medical history. To investigate tumour staging Total Body CT scan was performed confirming the presence of enlarged cervical and abdominal lymph nodes with typical patterns of malignancy. Bone scintigraphy and bone marrow biopsy were negative. Brain MRI, lumbar puncture and cerebrospinal fluid cytology showed any involvement of central nervous system (CNS). However due to the presence of third branch trigeminal nerve palsy CNS involvement was considered. Staging classification was Ann Arbor Stage IV.

The patient was treated according to the Italian Association of Pediatric Hematology Oncology (AIEOP) protocol which includes dexamethasone, ifosfamide, methotrexate, cytarabine, etoposide phosphate, vincristine, cyclophosphamide, daunomycin and intrathecal administration of prednisolone, cytarabine and methotrexate. He received four courses of chemotherapy resulting in a rapid tumor regression followed by the complete remission (CR) after the fourth course. The neutropenic period lasted from day 7 to day 12 after treatment without further chemotherapic toxicity. During this period he received antibacterial, antiviral and antifungal prophylaxis as well as blood transfusions and G-CSF support. In parallel with chemotherapy, highly active antiretroviral therapy (HAART) based on four drugs (lopinavir/ritonavir + abacavir/lamivudina) was started.

The patient, over the last 3 years, has been regularly followed-up by the HIV Unit of our Hospital. Blood count, LDH, EBV, HIV viral load and CD4 cell counts were detected every 3 months. Abdominal and cervical ultrasound were performed at regular intervals of six months. Viral load resulted undetectable (number of copies <50/ml) after 10 months of HAART initiation. The patient remains in good health condition maintaining CR over the last 3 years of follow-up. HIV plasma viral load is still undetectable with good immune reconstitution (CD4 count 603/mm3 or 24,5%) [Table 1].

## Discussion

We report a case of vertical HIV infection presenting with atypical features because of the timing of presentation and the long term period free of the characteristic early signs and symptoms of AIDS.

Human immunodeficiency virus (HIV)-infected children have an increased risk of developing cancer, particularly in the later stages of acquired immune deficiency syndrome (AIDS) and AIDS-related non-Hodgkin's lymphomas are one of the most common AIDS-defining malignancies [1].

This seems to be related to multiple factors, including the transforming properties of the human retrovirus itself, the duration and degree of immunodeficiency and viral exposure [2], the immune activation leading to B cell proliferation and further infections with other lymph tropic viruses such EBV [3,4].

Without Antiretroviral Treatment (ART), progression of HIV infection in vertically infected children is most rapid than in adults and about one-fifth progress to serious clinical illness or die before one year of age [5]. In fact, in HIV-1- infected non treated children the rates of progression to AIDS are 17% at 1 year of age and 35% at 5 years of age [6-8]. For this reason, BL is a common co-morbidity of HIV infection particularly in developing countries, where people do not have access to antiretroviral drugs [9], but rarely represents the initial clinical manifestation of vertical HIV infection in adolescent in Western Europe. Indeed, the introduction of protocols to prevent mother-to-child transmission and efficacious regimens of early antiretroviral treatment have resulted in a substantial and dramatic decrease in AIDS-related opportunistic infections and cancers in both adults and children [1,4,10-12].

However, despite this progress, recent data show as HIV infection itself represents a major risk factor for developing cancers [13] especially in young adults, a new phenomena made possible by the improved survival followed by HAART use and the low adherence to treatment reported during this age [14]. Within HIV-related tumors, Non Hodgkin's Lymphomas remain the most frequent malignancies of children with AIDS [15,16]. In the developed countries NHL accounts for 65%-83% of AIDS-related malignancies, and BL rates about 40% of HIV-related NHL in children [17,18]. Hence, BL rarely represents the first sign of HIV infection in childhood whose HIV exposure status is not known [19]. Interestingly in this case, although the patient vertically acquired HIV infection, he had an asymptomatic neonatal period and childhood, with normal growth and development, up to 16 years old. As previously discussed this silent pathological history led to misdiagnose the vertical HIV infection. However, the presence of marked hypergammaglobulinemia associated with severe immunodeficiency and the diagnosis of BL allowed us to suspect and subsequently diagnose HIV infection and start an adequate treatment.

Before the widespread use of HAART, in patients with BL and HIV, the chemotherapy was less aggressive than in non-HIV-infected patients, with parallel corresponding poorer results. This approach is presently abandoned, since recent cumulative evidences have showed comparable results with full intensive chemotherapy between HIV infected and non-infected individuals in terms of toxicity effects and survival [20]. Indeed, although the patient hereby described received high-dose polychemotherapy, no serious toxic effects neither life-threatening infections were recorded.

Patients who developed NHL but who did not undergo HAART had better survival, particularly when compared with patients who developed NHL after HAART initiation [21-23]. Thus, starting antiretroviral therapy in addition to chemotherapy after the diagnosis of NHL may represent an effective salvage therapy even for those patients who have NHL as their AIDS-defining event, as in this case [22,23].

## Conclusion

This case illustrates a HIV-associated BL, initially misdiagnosed as a common mononucleosis syndrome. It represents an atypical presentation of vertical HIV infection because of the total absence of characteristic early signs and symptoms of AIDS.

This case highlights the increased risk to develop NHL of young HIV positive individuals. Indeed, despite the introduction of antiretroviral therapy several studies report a lesser decrease in incidence rates of NHL compared with others type of cancers (i.e. Kaposi's sarcoma) in this population [13,24]. Recent data show as immunodeficiency and HIV viremia are major risk factors for developing cancers [2,13,24] especially in young adults. In this optic, lower adherence to treatment reported during this age [14] might represent an emerging problem. Thus, access to NLH screening programmes should be regularly offered to all HIV-positive adolescents and young adults [24].

## Consent

Written informed consent was obtained from the patient for publication of this case report and any accompanying images.

## Competing interests

The authors declare that they have no competing interests.

## Authors' contributions

VS: made substantial contributions to conception and design of case report.

AA: made contributions to acquisition and interpretation of data.

NM: has been involved in drafting the manuscript or revising it critically for important intellectual content.

HTC: has been involved in drafting the manuscript or revising it critically for important intellectual content.

GP: has given final approval of the version to be published.

SB: made substantial contributions to clinical management of the patient and has given final approval of the version to be published

RDV: has been involved in acquisition, analysis and interpretation of histological data.

PP: made substantial contributions to clinical management of the patient and has given final approval of the version to be published

All authors read and approved the final manuscript.
